# A receptor and chaperone that influences lung MAIT cell number, function, and lung inflammation

**DOI:** 10.21203/rs.3.rs-10632260/v1

**Published:** 2026-10-19

**Authors:** Mitchell Kronenberg, Gabriel Ascui, Eleni Phung, Alba Mendis, Cindy Luna, Priscilla Gonzalez, Kenneth Kim, Sara McArdle, Dominic Haas, Xiaoyan Wang, Jihye Han, Shilpi Chandra, Ann Canonigo-Balancio, Ting-Fang Chou, Michael Constantinides, Kathleen Caron

## Abstract

Innate-like T cells are tissue-resident lymphocytes, but the factors that govern their localization to specific tissues, at steady state or after infections, are not well defined. Mucosal-associated invariant T (MAIT) cells are an innate-like T cell population that is relatively frequent in the lungs of mice and humans. Receptor activity-modifying protein 3 (RAMP3) is a member of a family of three proteins that modulate the expression and function of several GPCRs, including those involved in immune responses. Despite this, the role of RAMP3 in T cell immunity has not been widely explored. We found that *Ramp3* ^–/–^ mice had increased lung MAIT cells at steady-state or after infection, while other T cell populations were less affected. *Ramp3* ^–/–^ mice had increased surface CXCR6 expression, and RAMP3 overexpression reduced CXCR6. CXCR6 was required for the dramatic increase in lung MAIT cells after infection. These data suggest that RAMP3 negatively regulated CXCR6 and limited lung MAIT cell numbers. This has important consequences, because infected *Ramp3* ^–/–^ mice had increased IL-17A-secreting lung MAIT cells, but were more susceptible to pulmonary infection, with reduced bacterial clearance, increased cellular infiltration, and immunopathology. The increase in lung MAIT cells after infection occurred predominantly from cells located in the tissue, but in *Ramp3* ^–/–^ mice there was evidence for some recruitment to the lung. We have established that RAMP3 is a negative regulator of excessive lung MAIT cell responses, acting in part through CXCR6.

